# Analytical Performances of the Panther Fusion System for the Detection of Respiratory Viruses in the French National Reference Centre of Lyon, France

**DOI:** 10.3390/microorganisms8091371

**Published:** 2020-09-07

**Authors:** Maxime Pichon, Martine Valette, Isabelle Schuffenecker, Geneviève Billaud, Bruno Lina

**Affiliations:** 1 Laboratoire de Bactériologie-Hygiène, CHU Poitiers, Département des Agents Infectieux, 86021 Poitiers, France; 2 Laboratoire de Virologie, Hospices Civils de Lyon, Institut des Agents Infectieux, Centre National de Référence des Virus des Infections Respiratoires (dont la Grippe), Hôpital de la Croix-Rousse, 103 grande rue de la Croix-Rousse, CEDEX 04, 69317 Lyon, France; martine.valette@chu-lyon.fr; 3 Laboratoire de Virologie, Hospices Civils de Lyon, Institut des Agents Infectieux, Centre National de Référence des Enterovirus et Paréchovirus, Hôpital de la Croix-Rousse, 103 grande rue de la Croix-Rousse, CEDEX 04, 69317 Lyon, France; isabelle.schuffenecker@chu-lyon.fr; 4 Laboratoire de Virologie, Hospices Civils de Lyon, Institut des Agents Infectieux, Hôpital de la Croix-Rousse, 103 grande rue de la Croix-Rousse, CEDEX 04, 69317 Lyon, France; genevieve.billaud@chu-lyon.fr

**Keywords:** respiratory viruses, influenza, diagnostic PCR, performance evaluation, automation, respiratory tract samples, sample-to-answer real-time PCR

## Abstract

Respiratory infection are mainly caused by viral pathogens. During the 2017–2018 epidemic season, Panther Fusion^®^ Respiratory kits (Influenza virus A&B (FluA&B), respiratory syncytial virus (RSV), adenovirus (ADV), metapneumovirus (MPV), rhinovirus (RV), parainfluenzae virus (PIV), were compared to the Respiratory MultiWells System r-gene. Respiratory clinical specimens were tested retrospectively (*n* = 268) and prospectively (*n* = 463). Analytical performances were determined (sensitivity –Sep-, specificity –Spe- and κ) considering concordances of ≥2 molecular testing specific to each viral target (discrepant results were verified at the National Reference Centres for Enteroviruses or Respiratory viruses, Lyon, France). After retrospective (and prospective) testing, Sep, Spe, and κ were 100% (97.7%), 100% (99%) and 100% (94%) for FluA: 100% (95.5%), 100% (99.3%) and 100% (94%) for FluB, and 100% (88.5%), 100% (98.7%) and 100% (89%) for RSV; 82.1% (41.7%), 100% (99.5%) and 86% (54%) for ADV; 94.7% (73.7%), 96.1% (98.0%) and 91% (65%) for MPV; 96.1% (94.6%), 90.2% (98.5%) and 86% (91%) for HRV; and 90% (72.7%), 100% (99.3%) and 91% (72%), respectively, for PIV. Analytical performances were above 85% for all viruses except for ADV, MPV and PIV, confirming the analytical performance of the Panther Fusion system, a high throughput system with reduced turn-around-time, when compared to non-automated systems.

## 1. Introduction

Acute respiratory tract infections (ARIs), mostly caused by viruses, are a common and major cause of morbidity and mortality worldwide [1,2,3,4]. Seasonal respiratory illnesses caused by influenza virus (FluA and B) and respiratory syncytial virus (RSV) are responsible for most of the hospitalizations (3 to 5 million severe cases annually) and mortality (290–600 thousand deaths annually) [5,6,7] but studies have reported other viruses that are associated with ARIs, such as parainfluenza viruses 1 to 4 (PIV 1 to 4), coronaviruses, metapneumovirus (MPV), rhinovirus (RV), adenovirus (AdV), and bocavirus [8,9]. With a wide spectrum of symptoms, diagnosis, when based on clinical presentation alone, is clearly limited and requires biological testing [10]. Considering microbiological diagnoses as critical for clinical management, accurate and timely identification of the pathogen is the key for optimized clinical management of the disease in order to administer appropriate antiviral therapy, adopt public health measures, and control outbreaks. Replacing classical virological culture, nucleic acid amplification testing (NAAT) has significantly reduced antibiotic use and length of hospital stay over recent years [11,12].

With the advent of multiplex real-time respiratory panels, diagnosis of multiple infections caused by respiratory pathogens is achieved with appropriate turnaround time for clinical decision-making [13,14,15]. On the fully automated Panther Fusion system, the Panther Fusion respiratory assays (Hologic Inc., San Diego, CA, USA) consist of three separated multiplex real-time PCR panels designed to detect an array of respiratory viruses. The turnaround time of this assay, with possible continuous loading, is evaluated at 2.5 h, with a throughput of up to 120 respiratory samples in an 8-h workday.

The present study aimed to compare the performance of the Panther Fusion Respiratory Virus and MultiWells System R-Gene panels on clinical respiratory samples in detection of influenza A and B viruses, RSV, PIV, MPV, RV and AdV.

## 2. Materials and Methods

### 2.1. Study Design and Sample Selection

The study was divided into two phases, both of which were conducted at the Virology Department of the Infectious Agents Institute (University Hospital of Lyon, Lyon, France).

For the retrospective phase, clinical samples were retrospectively and randomly selected (including nasal swab—NS, nasopharyngeal aspirate—NPA, tracheobronchial aspirate—TPA and bronchoalveolar lavage—BAL) in a sample biobank (five consecutive epidemic seasons i.e., 2012 to 2017) to include at least 30 samples (50 for PIV) per viral target. These samples were conserved frozen (−80 °C) until thawing and testing for the purposes of the study on the Panther Fusion System (Hologic).

For the prospective phase, clinical samples were prospectively selected over 15 consecutive weeks from November 2017 and March 2018. Thirty samples per week were included (one third of nasopharyngeal aspirates and two thirds of nasal swabs) from patients suspected of respiratory viral infection. All ages were included, respecting a proportion of one included child per included adult patient. The samples were not frozen before testing and were tested simultaneously by both assays in the same week.

### 2.2. Analytical Process

All the specimens were collected in Universal Transport Medium tubes (Copan Diagnostics, CA, USA) and were then tested in blinded fashion by two different operators in the Infectious Agents Institute (University Hospital of Lyon, France). One fresh aliquot was dedicated to be analyzed on the Panther Fusion System (Hologic) after routine clinical management of the respiratory sample (reference testing). A supplementary aliquot was stored frozen at −80 °C and dedicated to analysis in case of discrepant results.

For the Panther Fusion, three different diagnostic tests were used to detect different respiratory virus targets, according to manufacturer’s recommendations. Samples were transferred to a specimen lysis tube (according to manufacturer’s recommendations) and then loaded directly onto the Panther Fusion System (Hologic Inc., Marlborough, MA, USA), that performed automated nucleic acid extraction and amplification. Three different kits were applied for all samples i.e., 1/FluA, FluB and RSV; 2/PIV1, PIV2, PIV3 and PIV4; 3/AdV, MPV and RV.

The reference testing was performed according to respective manufacturer’s recommendations, using the MultiWells System (R-GENE MWS, bioMérieux, Marcy l’étoile, France), after automatic extraction using the EasyMag system (bioMérieux; 200 µL assay volume/50 µL elution volume), on an ABI7500 thermocycler (Applied Biosystems, Lifetechnologies, Carlsbad, CA, USA). All the assays were performed according to the respective manufacturers’ recommendations.

Both platforms allow visualization of the amplification curves and respective Cycle threshold (Ct) values. Interpretation was carried out following the manufacturer’s recommendations. The main characteristics of the two tests compared in this study (equipment, degree of automation, detection format, hands-on-time, primary tube utilization, detection throughput, number of reactions per run, turnaround time, volumes—sample, elution, PCR—targeted genes, presence/absence of controls, number of amplification cycles, conditioning and reagent storage temperature) are shown in Table 1.

### 2.3. Consensus Result and Discordant Resolution

In the case of discrepant results, results were verified on frozen-dedicated aliquots by the National Reference Center for Respiratory Viruses or on Enteroviruses and Parechoviruses, Lyon, France using an unpublished CDC RT-qPCR protocol for influenza viruses, a published RT-PCR for RSV, or a published protocol consisting in a semi-nested PCR followed by Sanger sequencing for RV [16,17]. No further evaluation, except for retesting, could be performed for MPV, PIV and AdV.

To evaluate diagnosis performances of the Panther Fusion, a consensus result was defined as a concordance of two molecular tests. For PIV, as the reference testing (R-GENE MWS) did not type these viruses, concordance was considered when PIV was detected by the two methods.

### 2.4. Statistical Analysis

Data analysis was performed using GraphPad Prism v6.0. A *p*-value of 0.05 was considered as significant. The 95% confidence interval (CI) was calculated for Sensitivity (Sep), Specificity (Spe) and Kappa coefficient (κ) using the Wald score method [18].

### 2.5. Ethical Statement

The study was approved by the Institutional Review Boards of the University Hospital of Lyon on 7 July 2017. In compliance with French law at the time of sampling, information was given to each patient consulting at the Hospices Civils of Lyon about the collection and use of biological samples for regular disease management and further epidemiological studies. For the purposes of this study, patient confidentiality was strictly protected, and samples were de-identified after routine management and before analyses.

## 3. Results

### 3.1. Retrospective Phase

For this phase, among the 268 samples tested and after exclusion of invalid samples (3 FluA/FluB/RSV; 1 AdV/MPV/RV; 2 PIV), 233 (233/268; 86.9%) were identified as consensus positive samples including 27 (100% of the positive strains) for Flu A, 27 (100% of the positive strains) for Flu B, 36 (36/36; 100%) for RSV, 36 (36/38; 94.7% of the tested strain) for MPV, 49 (49/51; 96.1%) for RV, 23 (23/28; 82.1%) for AdV, and 48 (48/51; 90.0% of the tested strains) for PIV. Among the tested samples, 26 samples (26/244; 10.7%) were positive for two different viruses (i.e., IAV/RSV, *n* = 2, 0.7%; AdV/RV, *n* = 12, 4.5%; AdV/MPV, *n* = 4, 1.5%; MPV/RV, *n* = 8, 3.2%) and one was positive with three different viruses (MPV/ADV/RV; 1/241; 0.4%). Both systems detected the dual infection, except for one sample positive for AdV and negative for RV in the Panther Fusion System. Results comparing each target/assay against the established consensus positive are shown in Table 2.

### 3.2. Prospective Phase

For this phase, in the tested samples (*n* = 463), 308 nasopharyngeal (308/463, 66.5%) and 155 nasopharyngeal aspirates (155/463, 33.5%) were included. Among them, 243 were pediatric samples (243/463, 52.5%), and 221 (220/463, 47.5%) were adult samples. Six samples were considered as invalid (four nasal swabs and two nasopharyngeal aspirates) after the first analysis by the Panther Fusion system (four fluid aspiration error and two clots) and then excluded from further analyses.

A total of 229 (229/457, 50.1%) consensus samples were identified for specimens among the 457 samples analyzed, including 183 single identifications (212/229; 92.6%), 16 double identifications (16/229, 7.0%) and one three-virus identification (1/229, <0.5%). Among the consensus samples, 43 Flu A (43/229, 18.8%), 42 Flu B (42/229, 18.3%), 54 RSV (54/229, 23.6%), 14 MPV (14/229, 6.1%), 53 RV (53/229, 23.1%), 15 AdV (15/229, 6.6%), and 8 PIV (8/229, 3.5%) were included. Results comparing each target/assay against the established consensus positive are shown in Table 3.

### 3.3. Investigation on Discrepant Results

For both prospective and retrospective phases, samples with discrepant results on IAV, IBV, RSV, and RV were further evaluated with the third methods approved by French National Reference Centers and were considered as low Ct value when Ct < 37 (high viral load) and high Ct value when Ct > 37 (low viral load). All discrepant Ct values are summarized in Table 4.

In the absence of a third method, all discrepant results implicating a PIV, MPV, or ADV were excluded from further investigation. Retesting using both the reference and tested methods was applied for the prospective phase to determine the estimated viral load. For PIV, the discrepant results demonstrated four high Ct values and two low Ct values. For ADV, the discrepant results demonstrated eleven high viral loads and twelve low Ct values. Finally, for MPV, the discrepant results demonstrated six high viral loads and eight low Ct values.

Among the samples of the prospective phase which remained discrepant, 25 presented low Ct value (i.e., medium-to-high viral load) (25/33, 75.8%): 13 false positive (13/25, 52.0%; FluA: 3/13 −23.1%-, FluB: 3/13 −23.1%-, RSV: 4/13 −30.8%-, and RV: 3/13 −23.1%-) and twelve false negative results (12/25,48.0%; FluA: 1/12 −8.3%-, FluB: 1/12 −8.3%-, RSV: 7/12 −58.3%-, and RV: 3/12 −25.0%-). Those with a high Ct value included seven false positive (7/8, 87.5%; FluA: 2/7 −28.6%-, FluB: 1/7 −14.3%-, RSV: 1/7 −14.3%-, and RV: 3/7 −42.9%-) and only one false negative sample (1/8, 12.5%; positive with RSV).

Among the samples of the retrospective phase which remained discrepant, only RV samples could be investigated (*n* = 7). The two false negative results (2/7, 28.6%, HRV-C) demonstrated high Ct value (34.5 and 35.0), while three false positive results implicated low Ct value (18.3, 28.7, and 33.8) and two high Ct values (40.3 and 42.2).

## 4. Discussion

In this study, the performances of the three Panther Fusion respiratory assays were evaluated and compared against well-validated assays and protocols. Since their introduction, there have been several studies focusing on the performance of molecular biology assays for detection of respiratory viruses. Approaches considered by these different tests included whole multiplex assays, or seasonal panels (limited to Flu with or without RSV), as different systems came on the market to fill particular needs in a specific clinical situation. It is notable that the present study evaluated clinical specimens sampled after 2014, year of the first description of A(H3N2) C163T mutation in M1 gene that limited sensitivities of detection for some NAATs [19,20].

The presented results demonstrate that both Panther Fusion and comparator technologies produced comparable results for detection of the viruses responsible for most of the viral respiratory infections, with slightly higher performances for the Panther Fusion respiratory assays [2,3,4]. Similar results have been described when comparing Panther Fusion system to seasonal panels (i.e., Cobas Influenza A/B test (cIAB, Roche Diagnostics, Indianapolis, IN, USA), Xpt (Cepheid, Carlsbad, CA, USA), wide-range panels (i.e., Filmarray respiratory panels 1.7 (RP, BioFire, Salt Lake City, UT, USA), Allplex respiratory panels (Seegene, Seoul, Korea), eSensor RVP (eSensor; Genmark Dx, Carlsbad, CA, USA), Lyra (Quidel, San Diego, CA, USA)) and by laboratory designed tests or sequencing [21,22,23,24]. It is important to note that in this study, similarly to others, false results for both the methods are associated with higher Ct values (corresponding to the lowest viral load in the tested samples).

In the present study, every type of respiratory sample (including nasopharyngeal swabs or aspirates, and bronchoalveolar lavages) were tested, resulting in a significant number of samples with detected co-infection. These co-infections are crucial to detect because of their association with a higher risk of lower respiratory tract infection (mostly requiring hospitalization), even if they do not lead to worse disease outcome (according to a recent Taiwanese study) [25]. The system demonstrates performance as satisfactory in these samples as in the whole cohort.

The main limitation of the study, is that, by design, samples with discrepant results were not tested by a third methodology for PIV, MPV, and ADV. This lack of confirmation does not allow us to conclude whether these observations are due to greater sensitivity of the Panther Fusion system/assays (or due to true false viral detection) or to higher specificity (or due to true false negative results). Nevertheless, reading of the literature suggests that the superior analytical sensitivity of the Panther Fusion system was demonstrated in limit of detection and endpoint dilution studies [22]. With artificial samples mimicking clinical specimens, one can imagine that the discrepant samples observed in the present study were true positive/negative samples and consider that the described performances were under-determined (when considering discrepant results with low viral load as correct). Even considering that false negative results implicating high Ct values remain, the latter would suggest that both the MWS assays and Panther Fusion system assays demonstrated similar qualitative results on samples that would be confirmed by a third methodology. This remains to be analyzed in a similar study focusing on ADV, MPV and PIV.

Recently, as demonstrated by focused multiplex/syndromic panels, there has been discussion on the economic benefit, and clinical impact (in the absence of specific treatment against viral pathogens), of wide-range multiplex respiratory panels, raising questions about the ordering of the biological tests. As a comprehensive approach has to be set up for all microbiological assays, particularly in virological testing (and especially in medium or low-income countries or regions), smaller (or split) respiratory panels could be of great interest. Indeed, this approach could allow the providers to be more flexible, limiting their investigation to one of the three panels, and possibly to enhance their diagnosis to a dual- or full-combination of the available assays, based on the epidemiological situation or the clinical presentation of the sampled patient.

## 5. Conclusions

In conclusion, the Panther Fusion respiratory assays performed with similar positive and negative predictive agreements to the MultiWells System r- Gene for most of the targets tested. This system provides laboratories with a system to test for a broad array of viral respiratory pathogens, allowing a fully automated RT-PCR process and random access with clinically appropriated turnaround time to be implemented in routine clinical viral diagnostics medium to high throughput clinical labs.

## Figures and Tables

**Table 1:** Main characteristics of compared systems.

Parameter	MultiWells System r-Gene			Panther Fusion
Equipment	Validated extraction platform: NUCLISENS^®^ EASYMAG^®^ MagNA Pure Compact QIAsymphony SP	Validated amplification platform: LightCycler 480 System II RotoR-GENE Applied Biosystems 7500 Fast, StepOne Stratagene/Agilent/VERSANT kPCR Molecular System AD Dx Real-Time System		Specific all-in-one platform
Automation	Include amplification and analysis			Include extraction, amplification and analysis
Amplification platform	Multiplex one-step RT-PCR			
Detection Format	Real-Time PCR/5′ nuclease Taqman technology			
Hands-on-time (min)	20 (for 96 samples maximum)		20	
Primary tube utilization	Yes		No	
Detection throughput	Batches		Random Access	
Number of reactions per run	Up to 96 tests at a time		60 tests at a time	
Test turnaround time (including analysis)	1.5 h (extraction step not included)		2.5 h (including extraction step)	
Needed sample volume (µL)	Depending on the platform used for extraction		500	
Produced elution volume (µL)	Depending on the platform used for extraction		50	
PCR reaction total volume (µL)	25 (including extract: 10)		25–30 (including extract: 5–10)	
Targeted gene	M gene (influenza, metapneumovirus (MPV)) N gene (respiratory syncytial virus (RSV), parainfluenzae virus (PIV) HEXON (adenovirus (AdV)) 5′ non-coding region (rhinovirus (RV)/EV)		M gene (influenza A (FluA), influenza B (FluB), RSV) HEXON (AdV) Hemagglutinin-neuraminidase (PIV1,PIV2,PIV3)—N gene (MPV, PIV4) 5′ non-coding region (RV)	
Controls included	Positive Control and Negative Control			
Reporting unit	Qualitative test			
PCR amplification cycles	40		45	
Number of assays per kit	60		96	
Reagent storage temperature	−18 °C/−22 °C		4 °C	

**Table 2:** Clinical sensitivity and specificity of Panther Fusion respiratory panels for the retrospective phase.

		Sample Nature (*n*; %)				Sensitivity			Specificity			Kappa Coefficient	
		NS	NPA	TBA	BAL	*n*	%	95% CI	*n*	%	95% CI	κ	95% CI
*n* = 94	FluA	29 (30.8%)	64 (68.1%)	1 (1.1%)	-	27/27	100	85.2–100	67/67	100	93.5–100	100%	100–100
	FluB					27/27	100	85.2–100	67/67	100	93.5–100	100%	100–100
	RSV					36/36	100	88.5–100	58/58	100	92.6–100	100%	100–100
*n* = 89	MPV	27 (30.3%)	57 (64.0%)	3 (3.4%)	2 (2.2%)	36/38	94.7	81.8–99.5	49/51	96.1	86.0–99.7	91%	82.0–99.6
	RV					49/51	96.1	86.0–99.7	46/51	90.2	78.6–96.2	86%	76.5–96.1
	AdV					23/28	82.1	63.9–92.6	61/61	100	92.9–100	86%	74.8–97.9
*n* = 74	PIV	41 (55.4%)	28 (37.8%)	1 (1.4%)	4 (5.4%)	48/51	90.0	83.5–98.6	23/23	100	83.1–100	91%	80.1–100

NS: nasal swab; NPA: nasopharyngeal aspirate; TBA: tracheobronchial aspirate; BAL: bronchoalveolar lavage.

**Table 3:** Clinical sensitivity and specificity of Panther Fusion respiratory panels for the prospective phase.

	Sensitivity			Specificity			Kappa Coefficient	
	*n*	%	95% CI	*n*	%	95% CI	κ	95% CI
FluA	43/44	97.7	87.1–99.9	409/413	99.0	97.4–99.7	94%	88.6–99.2
FluB	42/44	95.5	84.0–99.6	410/413	99.3	97.8–99.9	94%	88.4–99.2
RSV	54/61	88.5	77.9–94.6	391/396	98.7	97.0–99.6	89%	82.1–94.9
MPV	14/19	73.7	50.9–88.6	429/438	98.0	96.1–99.0	65%	48.0–82.1
RV	53/56	94.6	84.8–98.7	395/401	98.5	96.7–99.4	91%	85.3–96.8
AdV	15/36	41.7	24.1–57.8	419/421	99.5	98.2–99.9	54%	38.0–70.6
PIV	8/11	72.7	42.9–90.8	443/446	99.3	97.9–99.9	72%	50.7–93.5

**Table 4:** **Ct values of discrepant results.** IQR: inter quartile range; -*: data not calculated (one value only); NA: not calculated (no values). IAV: influenza A virus; IBV: influenza B virus; RSV: respiratory syncytial virus; ADV: adenovirus; MPV: metapneumovirus; RV: rhinovirus; PIV; parainfluenzae Virus.

Viral Target	Prospective Phase		Retrospective Phase	
	False Detection (Median; IQR)	Absence of Detection (Median; IQR)	False Detection (Median; [IQR])	Absence of Detection (Median; [IQR])
**IAV**	35.1 (25.7–38.5)	29.3 (-*)	NA	NA
**IBV**	34.8 (32.1–37.2)	30.4 (30.3–30.6)	NA	NA
**RSV**	36.0 (35.8–36.0)	33.4 (20.0–35.0)	NA	NA
**ADV**	27.3 (22.0–32.6)	37.2 (36.2–37.8)	NA	37.4 (36.1–38.0)
**MPV**	40.3 (37.2–41.6)	31.4 (29.4–33.8)	40.2 (39.8–40.7)	33.3 (32.3–34.2)
**RV**	38.1 (34.7–40.4)	23.7 (23.2–26.2)	33.8 (28.7–40.3)	34.7 (34.6–34.9)
**PIV**	36.1 (35.6–39.1)	33.0 (28.7–37.7)	NA	30.5 (27.1–34.8)
